# Pleomorphic xanthoastrocytoma with multiple recurrences and continuous malignant progression to bone metastasis: a case report

**DOI:** 10.3389/fsurg.2025.1595199

**Published:** 2025-06-04

**Authors:** Lei Tian, Wei Sun, Lei Lou, Wenyan Wang, Yanan Li, Huandi Zhou, Zhiqing Xiao, Xiaoying Xue

**Affiliations:** ^1^ Department of Radiotherapy, The Second Hospital of Hebei Medical University, Shijiazhuang, Hebei, China; ^2^ Department of Pathology, The Second Hospital of Hebei Medical University, Shijiazhuang, Hebei, China

**Keywords:** polymorphic xanthoastrocytoma, bone metastasis, poor prognosis, BRAF V600E mutation, case report

## Abstract

Pleomorphic xanthoastrocytoma (PXA) is a rare benign WHO grade II astrocytoma predominantly observed in pediatric and adolescent populations, with a higher incidence in superficial brain regions. Histologically, PXA is distinguished by pleomorphic cells, lipidized cells, and eosinophilic granular bodies, frequently associated with BRAF V600E mutation and homozygous deletion of CDKN2A/B. While the overall prognosis for PXA patients is favorable, a subset of cases may progress to anaplastic PXA (WHO grade III astrocytoma) or undergo malignant transformation into epithelioid glioblastoma (E-GBM, WHO grade IV astrocytoma). The latter condition is characterized by BRAF V600E mutation, TERT promoter mutation, and aggressive clinical behavior, although distant metastasis remains uncommon. This case report describes a rare and complex malignant transformation and systemic metastasis of PXA. A 14-year-old male was diagnosed with right frontal-parietal PXA (WHO grade II astrocytoma) in 2011. After surgery, there was no recurrence for nine years. In 2020, the tumor recurred as high-grade glioblastoma, with primitive neuroectodermal and spindle cell sarcoma components. Molecular analysis revealed BRAF V600E mutation and CDKN2A homozygous deletion. Despite multiple treatments including surgery, radiotherapy, and targeted therapy, the tumor continued to progress. By 2024, the disease had spread to the spinal cord and bones, leading to the patient's death. The complex molecular mechanisms of PXA's malignant transformation require an optimized targeted therapy approach based on molecular profiling and long-term vigilance for distant metastasis, guiding the management of similar cases.

## 1 Introduction

Pleomorphic xanthoastrocytoma (PXA), is a rare benign primary central nervous system (CNS) tumor classified as a WHO grade II astrocytoma, belongs to the astrocytoma subtype within neuroepithelial tumors. PXA is characterized by its favorable prognosis and predominantly affects children and adolescents, with no significant sex predilection. PXA originates from the special astrocytes located beneath the pia mater, accounting for less than 1% of all astrocytomavv (1). Classic PXA is characterized by distinct histological features and relatively indolent biological behavior. However, approximately 15%–20% of PXA cases exhibit anaplastic features, including prominent mitotic activity and/or necrosis. In the 2016 WHO Classification of Central Nervous System Tumors (4th edition), PXA with high mitotic activity or necrosis was designated as anaplastic pleomorphic xanthoastrocytoma, recognized as a distinct WHO grade III entity (2, 3). Notably, the 2021 WHO Classification (5th edition) eliminated the “anaplastic” terminology, unifying these tumors under the designation of PXA within the category of circumscribed gliomas. Current classification systems stratify PXA into WHO grades II and III based exclusively on histological criteria (4).

PXA is most frequently observed in the superficial regions of the brain, particularly in the temporal lobe, followed by the frontal and parietal lobes. Additionally, rare locations such as the cerebellum, thalamus, sella turcica, and brainstem have also been reported in isolated cases (5, 6). Clinical manifestations include headaches, seizures, sensory abnormalities (focal neurological deficits), nausea, and vomiting (secondary to elevated intracranial pressure) (7). On magnetic resonance imaging (MRI), PXA typically presents as a well-demarcated, round or irregular mass in superficial temporal regions. The tumors may present as solid, mixed cystic-solid, or purely cystic masses, with cyst walls frequently harboring mural nodules that are typically localized near the meningeal surface. Radiologically, these tumors demonstrate contrast-enhancing solid nodules, frequently accompanied by eccentric peripheral cystic components, perilesional edema, and occasional hemorrhage (8, 9). The fifth edition WHO classification of gliomas specifies that a definitive histopathological diagnosis of PXA requires the identification of astrocytic tumors with pleomorphic cellular components, including large multinucleated cells, spindle cells, and lipidized cells, frequently accompanied by eosinophilic granular bodies (10). Ideal diagnostic criteria further encompass pericellular reticulin deposition, BRAF mutations or MAPK pathway alterations, CDKN2A/B homozygous deletions, and a PXA-specific DNA methylation profile. BRAF alterations in PXA predominantly include gene fusions and BRAF V600E missense mutations, with BRAF V600E prevalence ranging from 50% to 78% in PXA and 47.4% in anaplastic variants. Primary treatment involves surgical resection, supplemented by adjuvant radiotherapy and chemotherapy. Despite its rarity, PXA generally exhibits favorable prognosis following gross total resection (11).

Epithelioid glioblastoma (E-GBM) is a rare variant newly classified under IDH-wildtype GBM (WHO grade IV astrocytoma) in the 2016 WHO schema. E-GBM is characterized by epithelioid and melanoma-like cells displaying abundant cytoplasm, eccentric nuclei, prominent nucleoli, and rhabdoid features (12). Distinct from conventional GBM, E-GBM frequently harbors BRAF V600E mutations, TERT promoter mutations, and CDKN2A/B homozygous deletions, correlating with aggressive clinical behavior, rapid recurrence, and dismal prognosis (13). At the epigenetic and cytogenetic levels, E-GBM shares the closest molecular resemblance to PXA. Clinically, patients with anaplastic PXA (A-PXA) may experience multiple recurrences, ultimately progressing to E-GBM with BRAF V600E mutations (10).

Malignant progression from low-grade pleomorphic PXA to anaplastic PXA and even high-grade neoplasms with complex histopathological components is exceedingly rare in clinical practice. This case demonstrated malignant transformation of PXA into a high-grade neuroepithelial tumor, notably progressing to a neoplasm featuring primitive neuroepithelial differentiation, focal spindle cell sarcomatous components, and characteristic genetic alterations—including BRAF V600E mutation and CDKN2A homozygous deletion. Furthermore, the patient experienced multiple recurrences and underwent extensive therapeutic interventions, including surgery, radiotherapy, and combination chemotherapy regimens. Ultimately, the disease metastasized to the spinal canal with widespread osseous dissemination, a phenomenon rarely documented in literature. This report provides novel insights into the diagnosis, management, and prognostic evaluation of such complex cases. Additionally, it offers clinical evidence to elucidate the transformation mechanisms linking PXA, A-PXA, and E-GBM.

## 2 Case presentation

**Figure 1:**
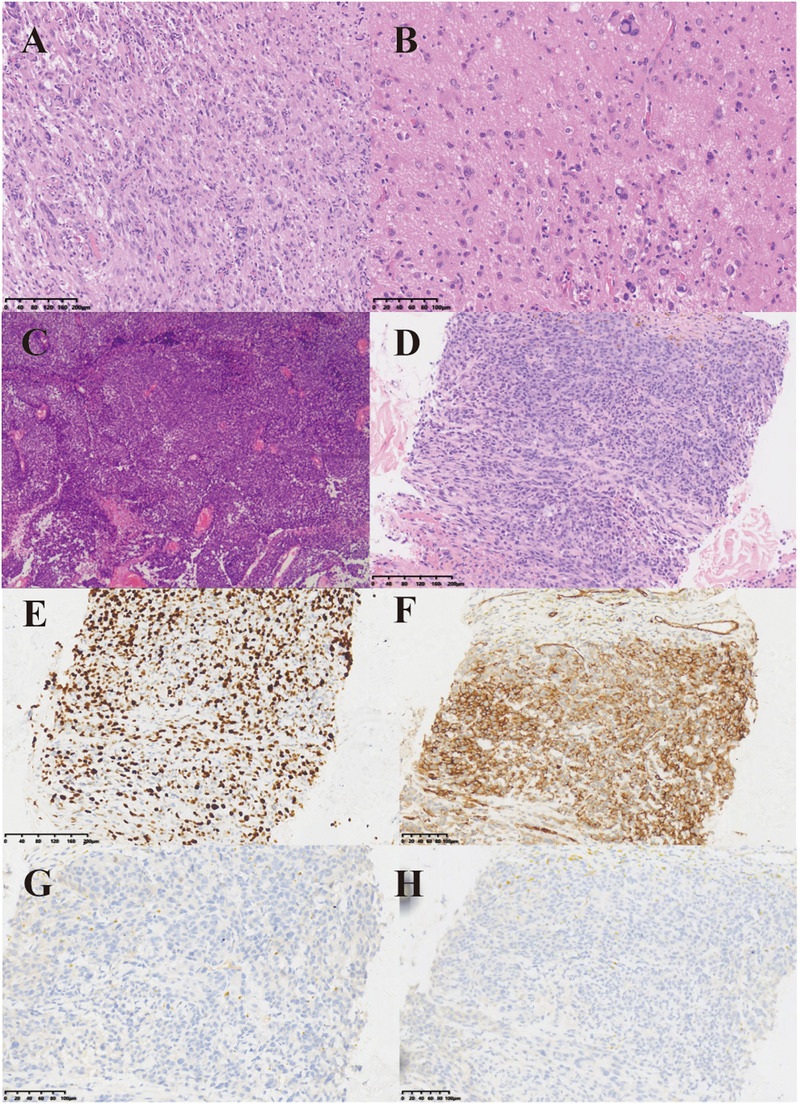
Histopathological features of tumors and metastases. **(A)** Under the low-power microscope, the postoperative pathology of the patient showed that the cytoplasm was eosinophilic, with visible cytoplasmic vacuoles, and the tissue density was relatively high. **(B)** Under the high-power microscope, the postoperative pathological examination of the patient showed diverse cell morphology. Large tumor giant cells and eosinophilic granular bodies could be observed, which are typical manifestations of PXA pathology. **(C)** HE staining of the patient's second postoperative pathology. Glioblastoma with primitive neuroepithelial and focal spindle cell sarcoma components. **(D)** HE staining of paraspinal metastases. HE staining revealed multifocal tumor components, with some cells exhibiting a spindle shape and visible mitotic figures. **(E–H)** Immunohistochemical staining of paraspinal metastases. Figure E to Figure H showed the expression of Ki-67, CD34, Oligo-2 and GFAP, respectively.

A 14-year-old male patient presented to our hospital in November 2011 with a history of intermittent headaches over several weeks. Cranial MRI revealed a space-occupying lesion in the right frontoparietal region. The patient underwent initial surgical resection, with histopathology confirming PXA (WHO grade II astrocytoma) (Figures 1A,B). Postoperatively, the patient underwent conventional radiotherapy and six cycles of temozolomide chemotherapy. Serial MRI surveillance over the subsequent nine years revealed no signs of recurrence.

**Figure 2:**
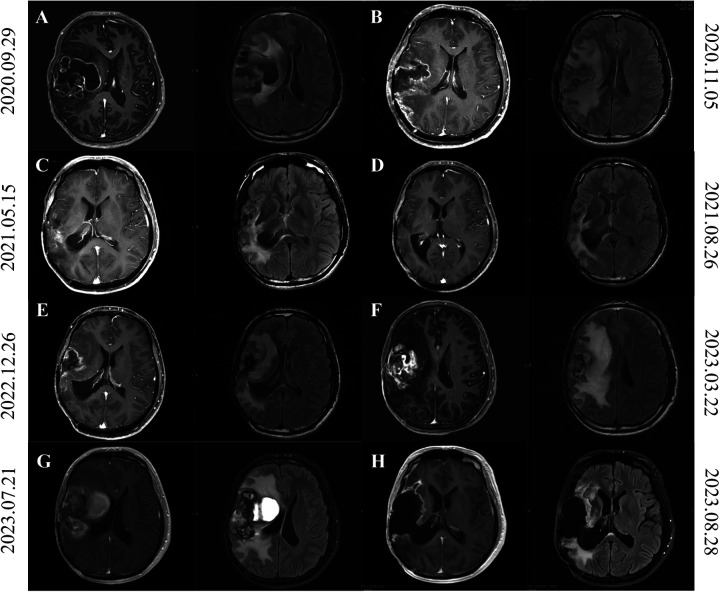
Brain MRI of patients from second tumor recurrence to third recurrence after surgery. **(A)** On September 29, 2020, MRI imaging demonstrated postoperative alterations in the right frontotemporal lobe, indicative of potential tumor recurrence. **(B)** MRI on November 5, 2020 showed a reduction in the size of lesions in the right frontotemporal lobe, a reduction in wall nodules, and a thickening of the meninges compared to MRI on September 29, 2020. **(C,D)** The cranial MRI on May 15, 2021 **(C)** and August 26, 2021 **(D)** was shown, showing that the tumor had progressed. **(E,F)** MRI of the head on March 22, 2023 **(F)**, compared with the images on December 26, 2022 **(E)**, showed slight enlargement of the right front parietotemporal lacunar after surgery and progressive deterioration of perifocal edema. **(G)** Figure shows the craniocerebral MRI findings in the presence of a tumor-derived hemorrhagic stroke. **(H)** This shows the postoperative craniocerebral MRI findings on August 23, 2023.

In April 2020, the patient experienced unexplained numbness and weakness in the left upper limb as well as facial numbness on the left side. These symptoms persisted for six months without significant improvement. Additionally, the patient reported intermittent headaches that did not respond adequately to ibuprofen and other analgesics. On September 29, 2020, an MRI revealed postoperative changes in the right frontal-temporal lobe, indicative of tumor recurrence (Figure 2A). Subsequently, the patient underwent a second surgical resection on October 10, 2020. Postoperative pathology confirmed a high-grade glioblastoma with primitive neuroepithelial and focal spindle cell sarcoma components (Figure 1C), characterized by BRAF V600E mutation, WHO grade IV astrocytoma, and MGMT promoter methylation.

Postoperative temozolomide chemotherapy was initiated but left limb numbness was not relieved. The patient was transferred to radiotherapy department for treatment. Contrast-enhanced MRI (November 5, 2020) showed reduced lesion size in the right frontotemporal lobe compared to prior imaging (September 29, 2020), with diminished mural nodules and meningeal thickening (Figure 2B). Postoperative radiotherapy (50Gry/25f) targeting the surgical cavity and T1-enhancing regions was administered concurrently with temozolomide, completed without adverse effects. The patient was advised to adhere to scheduled cranial MRI surveillance post-discharge and to seek medical attention in time if there is headache or epilepsy.

The patient underwent a follow-up head MRI at the outpatient department on May 15, 2021 (Figure 2C). Compared with the head MRI on March 17, 2021, the edema was slightly reduced by 5% this time, while the enhancement increased by 85% compared to before. According to the RANO criteria, tumor progression is considered. On August 26, 2021, a follow-up head MRI was conducted. Compared with the result of May 15, 2021, the peritumoral edema showed little change, but the range of enhancement increased, and the perfusion in the right surgical area decreased (Figure 2D). The cranial MRI indicated a neoplastic lesion. Given the intracranial progression, the patient was initiated on bevacizumab combined with temozolomide. Following six cycles of outpatient temozolomide chemotherapy, routine surveillance imaging was advised.

In March 22 2023, follow-up cranial MRI compared to prior imaging (December 26, 2022) revealed mild expansion of the right front parietotemporal postoperative cavity, marked enlargement of peripheral patchy enhancement, and progressive perilesional edema (Figures 2E,F). After a multidisciplinary consultation for neuro-oncology in our hospital, it was considered that the tumor had progressed, but the patient did not receive timely treatment.

On July 21, 2023, the patient visited our hospital due to a headache that had persisted for one week and was progressively worsening. The patient's headache was severe. After dehydration and intracranial pressure reduction treatment, the symptoms did not improve. An MRI of the brain showed a patchy slightly high-density shadow on the lateral side of the right frontal, parietal and temporal lobes, approximately 2.8*5.1 cm in size, suggesting partial hemorrhage in the brain tissue (Figure 2G). A mass in the right brain tissue was considered a recurrence of the tumor. There was a large area of low density in the right brain tissue, brain tissue swelling, the right ventricle was compressed and narrowed, and the midline structure was shifted to the left by about 0.8 cm. On July 22, 2023, the patient underwent a recurrence glioma resection through an expanded approach at the original incision on the right frontotemporal-parietal region. Postoperative management included antiedema measures and seizure prophylaxis, culminating in clinical stabilization and discharge on August 14, 2023. Histopathological evaluation of the third resection specimen confirmed a high-grade neuroepithelial tumor in the right frontal lobe, exhibiting microvascular proliferation, piecemeal necrosis, and hemosiderin deposition. Molecular pathology revealed BRAF V600E mutation and homozygous deletion of CDKN2A. The integrated diagnosis was anaplastic PXA. The images of two weeks after surgery were shown in Figure 2H. Adjuvant maintenance therapy with temozolomide and semustine was initiated postoperatively.

**Figure 3:**
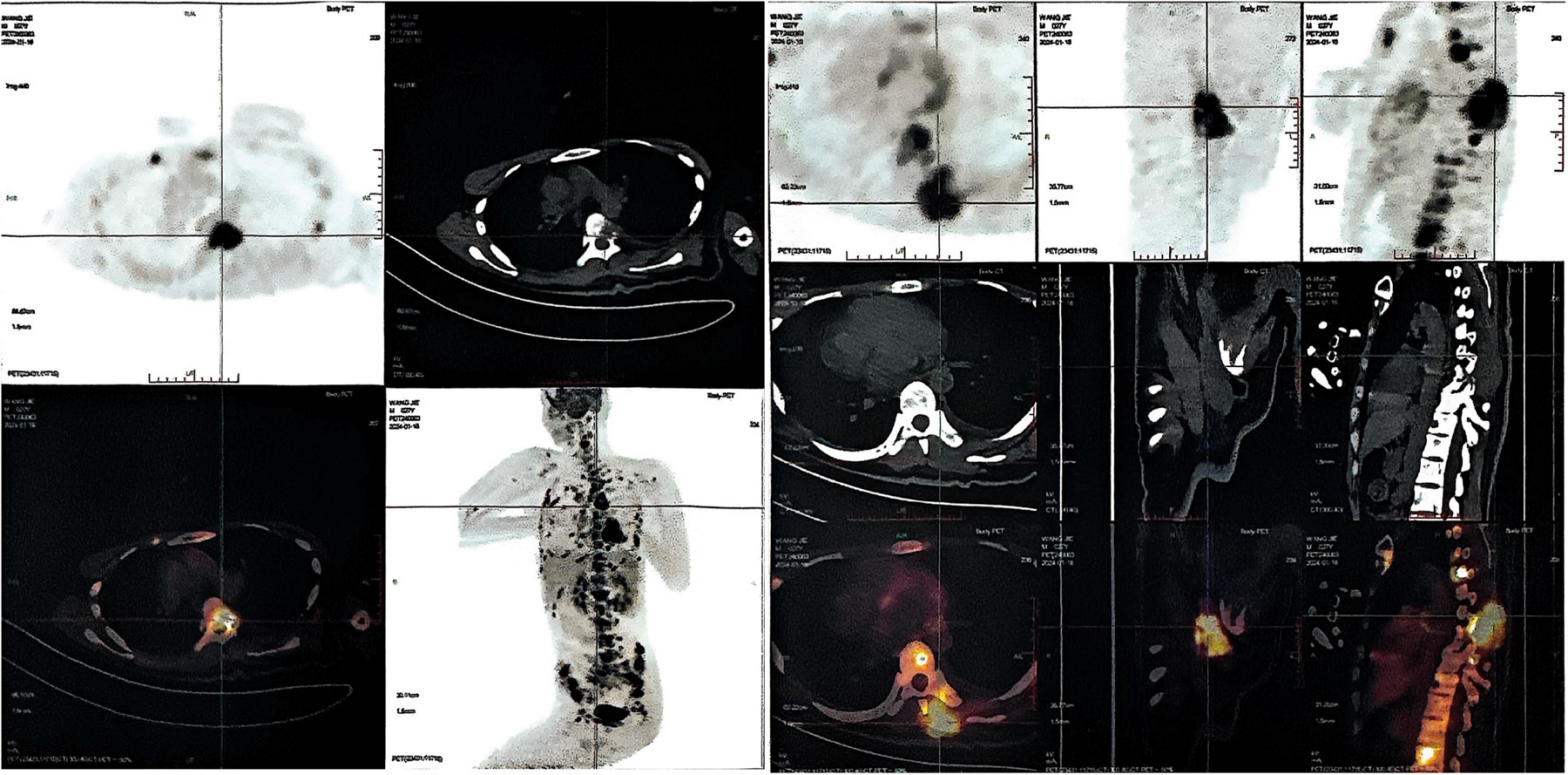
Positron emission tomography-computed tomography (PET-CT) imaging in patients with advanced stage tumors. Whole body PET-CT imaging showed multiple bone metabolic abnormalities, most notably in the spine, bilateral ilium and bilateral ribs.

**Figure 4:**
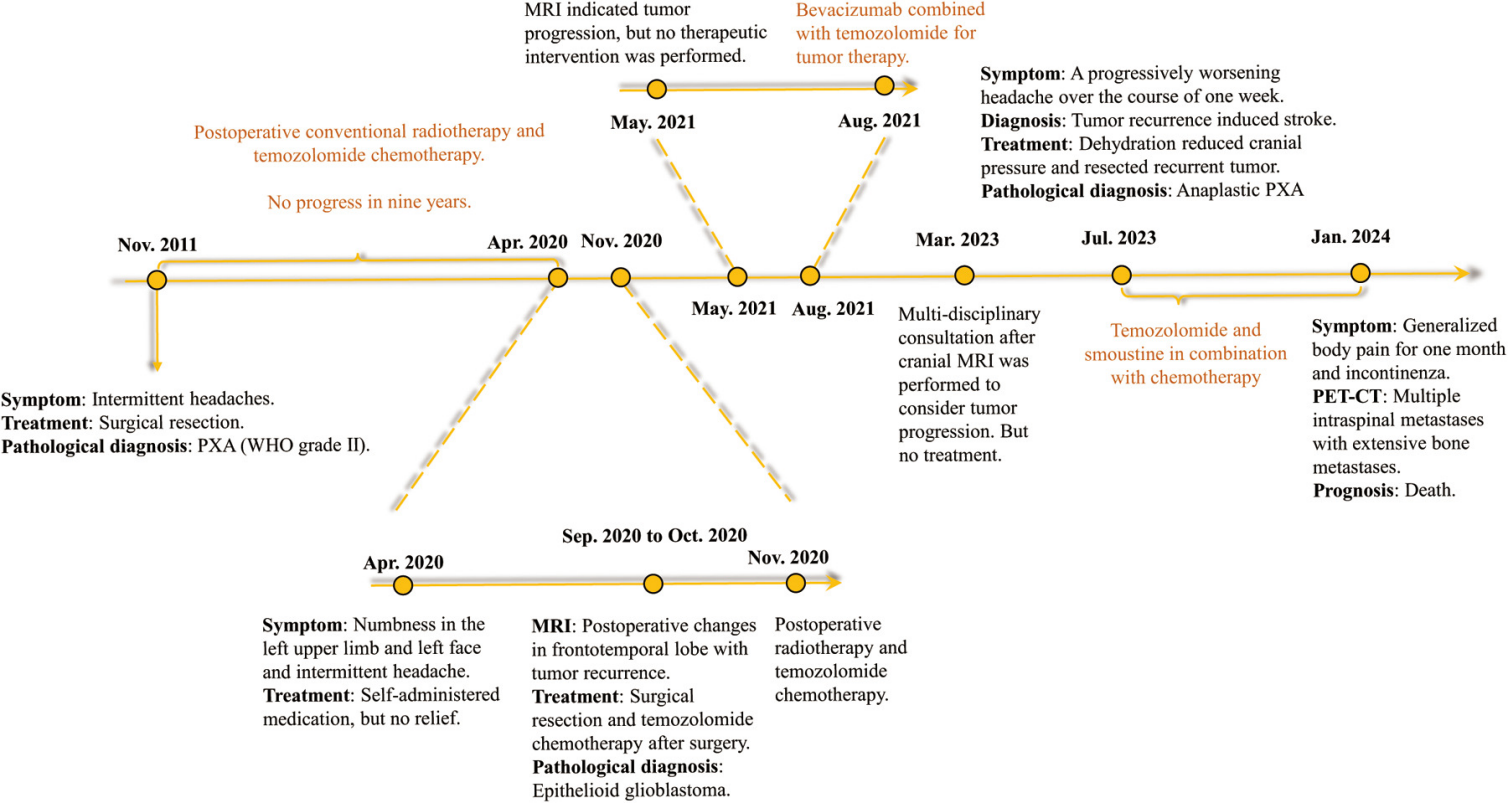
A timeline of the patient's entire course of illness from first onset to death.

On January 22, 2024, the patient suffered from body pain for 1 month and incontinenza. PET-CT examination showed that there were a few high-density shadows in the right parietal lobe, which could not rule out a few tumors' active lesions, cerebral parenchyma atrophy, and ventricle dilation. Multiple intraspinal metastases with extensive bone metastases (Figure 3). Biopsy of paravertebral masses was performed on January 23, 2024. Pathological diagnosis revealed multi-focal tumor components, some cells were fusiform, some nuclei were dislocated like rhabdomyoid, pseudo chrysanthemum cluster structures around blood vessels were visible, division images were visible (Figure 1D), and the immunophenotype was consistent with that of the original tumor. The results of immunohistochemistry are as follows: GFAP(-), Ki-67(70%+) Oligo-2(-), CD34(+) (Figures 1E–H). Later, the patient could not tolerate further anti-tumor therapy, and multiple organs gradually failed, leading to death. The entire course of the patient's disease is shown in Figure 4 in the form of a timeline.

## 3 Discussion

PXA is a very rare astroglioma, and PXA (WHO grade II) may undergo malignant transformation. According to relevant case reports, patients with PXA can malignantly transform into epithelioid high-grade glioblastoma (E-GBM), which is pathologically characterized by abundant epithelioid and melanoma-like cells with abundant cytoplasm, nuclear asymmetry, prominent nucleoli, and rhabdoid protein. PXA can also transform into anaplastic PXA. Anaplastic PXA is pathologically characterized by increased cellularity, increased pleomorphism and mitotic activity, necrosis and endothelial proliferation, which promote the clinical process of malignant progression of anaplastic PXA (13).

The uniqueness of this case lies in its complex course of disease development and rare pathological features. First, the patient had an initial diagnosis of PXA (WHO grade II) and had been in stable disease for 9 years after surgery, radiation therapy, and chemotherapy. However, it subsequently recurred and transformed into a high-grade neuroepithelial tumor, which was pathologically diagnosed as glioblastoma with primitive neuroepithelial and focal spindle cell sarcoma components. This transformation from low-grade to high-grade tumor is rarely reported in the literature. The mechanism of this transformation is unknown and may involve multiple molecular biological alterations. It is known that BRAF gene mutation plays an important role in the occurrence and development of some gliomas. In this case, BRAF V600E mutation persisted in different stages of the tumor, suggesting that BRAF V600E gene mutation may play a key role in the progression of the tumor. In addition, homozygous deletion of CDKN2A may also be associated with malignant transformation of tumors. CDKN2A and CDKN2B are tumor suppressor genes that play an important role in physiological processes such as cell division and growth. By regulating the cell cycle, cells can divide and proliferate in an orderly and controlled manner, and can inhibit the unlimited proliferation of cells (14). Mutations in CDKN2A and CDKN2B can lead to uncontrolled cell proliferation and even tumor growth. At present, mutations and deletions of CDKN2A and CDKN2B have been found in various tumors, and are associated with poor prognosis of tumors (15, 16). In glioblastoma, this gene mutation can also cause insensitivity or even resistance to radiotherapy and chemotherapy (17–19). Due to genetic mutations, many new targeted treatment strategies have emerged currently. Eric Bouffet et al. (20) demonstrated in a phase II clinical trial that for children with low-grade gliomas with BRAF V600 mutations, the combination of dabrafenib and trametinib as the first-line treatment resulted in more responses, longer progression-free survival, and better safety compared to standard chemotherapy. Francesca Del Bufalo et al. (21) treated 7 children with low-grade glioma who had BRAF V600E mutations with vemurafenib, and it was shown that the condition of 5 of the children was controlled. This indicates that BRAF/MEK can serve as an important drug target for glioma cells.

Next, patients exhibit complex radiographic and clinical features over multiple treatment sessions. Radiotherapy and chemotherapy are important means of comprehensive treatment for glioma, but in the present case, the tumor recurred and progressed multiple times despite standard treatment regimens. From the results of imaging examination, the performance of the tumor varies with each recurrence, such as edema, enhancement range, nerve fiber changes, and perfusion, which brings great challenges to clinical treatment decisions. For example, dynamic changes in enhancement and edema require a combination of considerations when evaluating tumor progression according to RANO criteria (22), whereas the complex changes in these measures in this case reflect tumor heterogeneity and variable response to therapy.

In addition, the patient eventually developed multiple intraspinal metastases with extensive bone metastases, which is very rare in patients with glioma (23). Glioma is usually characterized by local invasion, and distant metastasis is relatively rare, especially metastasis to the spinal canal and skeletal system. This phenomenon suggests the need to be alert to the possibility of distant metastasis in patients with recurrent glioma, even in the absence of significant extra-neurological symptoms. On the one hand, the change of biological characteristics of tumor cells is one of the key factors. In the process of tumor development, some tumor cells may acquire stronger invasion and migration ability, which can break through the restrictions of basement membrane and surrounding tissues and enter the blood circulation or lymphatic circulation system (24, 25). On the other hand, the destruction of the blood-brain barrier may also provide an opportunity for the metastasis of tumor cells (26, 27). The blood-brain barrier is originally a protective mechanism to prevent harmful substances from entering the brain tissue, but during the process of tumor growth, the blood-brain barrier may be damaged, making it easier for tumor cells to enter the blood circulation, thereby increasing the risk of distant metastasis. In addition, the decrease of immune function may also play an important role in tumor metastasis (26). Under normal circumstances, the body's immune system can recognize and eliminate tumor cells, but in the course of long-term disease and treatment, the patient's immune function may be suppressed, making it easier for tumor cells to escape the body's immune surveillance and form metastases in distant organs.

The course of treatment in this case also provides important lessons for clinical practice. Although a combination of surgery, radiotherapy, and chemotherapy is the current standard treatment option for gliomas, more individualized treatment strategies may be needed for patients with this complex condition (28). For example, in the case of tumor recurrence and poor response to conventional treatment, the treatment regimen of bevacizumab combined with temozolomide (29), although it controlled the tumor to some extent, still failed to stop the tumor progression. This suggests that we need to explore new therapeutic targets and drugs to improve the treatment effect of this rare and complex tumor. According to the gene mutation spectrum of tumor cells, appropriate targeted drugs are selected for precise treatment. In addition, the further improvement of multidisciplinary comprehensive treatment mode is also crucial. The close cooperation between neurosurgeons, radiotherapy doctors, chemotherapy doctors, pathologists and radiologists can develop a more comprehensive and individualized treatment plan for patients, improve the treatment effect and prognosis of patients. Due to the large time span of this study, some data are not retained, which is a great pity, but the only data can provide valuable experience for the management of this disease.

## 4 Conclusion

This case report describes a male patient was diagnosed with PXA (WHO grade II astrocytoma) after surgery at the age of 14 years. The course of disease spans 13 years, underwent three surgeries, multiple chemoradiotherapy, and targeted therapy, and finally died of tumor recurrence and extensive intraspinal and bone metastases. It is characterized by rapid tumor progression at later stages, a rare pattern of recurrence, and resistance to therapy, which are relatively rare in similar published cases. The complex treatment response, together with the rare occurrence of distant metastasis, presents a unique case in the field of neuro-oncology. In-depth study of such cases will help us better understand the biological behavior and pathogenesis of gliomas, and provide reference for the development of more effective treatment strategies in the future.

## Data Availability

The original contributions presented in the study are included in the article/Supplementary Material, further inquiries can be directed to the corresponding author.
